# De Novo Protein Design Enables Targeting of Intractable Oncogenic Protein–Protein Interfaces

**DOI:** 10.3390/biologics6010009

**Published:** 2026-03-18

**Authors:** Varshika Ram Prakash, Yusuf Najy, Kalel Garrett, Brian F. P. Edwards, Benjamin L. Kidder

**Affiliations:** 1 Department of Oncology, Wayne State University School of Medicine, Detroit, MI 48201, USA; 2 Karmanos Cancer Institute, Wayne State University School of Medicine, Detroit, MI 48201, USA; 3 Department of Biochemistry, Microbiology and Immunology, Wayne State University, Detroit, MI 48201, USA

**Keywords:** de novo protein design, computational binder design, protein–protein interactions, oncogenic protein interfaces, hotspot mapping, Molecular Operating Environment (MOE), AlphaFold2, immuno-oncology, therapeutic biologics

## Abstract

Background/Objectives: Protein–protein interactions (PPIs) involving oncogenic drivers remain among the most intractable targets in cancer biology due to their dynamic conformations and limited accessibility to conventional small molecules. Although antibodies and inhibitors have achieved clinical success against targets such as PD-1/PD-L1 and MYC, challenges persist related to tissue penetration, intracellular delivery, resistance, and incomplete blockade of key interface hotspots. The objective of this study is to develop an integrated computational framework for systematically designing hotspot-conditioned de novo miniprotein binders to target these interfaces. Methods: We present DesignForge, a computational protein design pipeline that integrates energetic hotspot identification, generative backbone design, sequence optimization, and structural confidence evaluation. The framework combines hotspot mapping using an open force-field-based energetic analysis module with generative backbone sampling using BindCraft, sequence optimization using ProteinMPNN, and structural validation using AlphaFold2. This in silico pipeline was applied to three representative oncogenic interfaces: PD-1/PD-L1, MYC/MAX, and KRAS/RAF. Results: Computationally generated designs exhibited high predicted structural confidence, favorable interface energetics, and consistent engagement of identified hotspot residues across targets. AlphaFold2-Multimer structural modeling indicated that the candidate PD-1 mimetic scaffolds, MYC/MAX interface binders, and KRAS interaction candidates can adopt conformations compatible with the target interfaces. Energetic contact analysis further supported predicted engagement of key hotspot residues. These findings support the computational feasibility of hotspot-conditioned binder generation using a unified design workflow. Conclusions: DesignForge provides a reproducible computational framework for hotspot-guided de novo protein binder design targeting oncogenic protein–protein interfaces. The designs reported here represent computational predictions derived from structural modeling and energetic analysis. Experimental biochemical and cellular validation will be required to determine the functional activity of the proposed binders.

## 1. Introduction

Protein–protein interactions (PPIs) orchestrate essential signaling, regulatory, and structural processes in cancer, yet many oncogenic PPIs remain refractory to conventional small molecules [1]. Unlike enzymes or receptors that contain deep, well-defined catalytic or allosteric pockets, most interaction surfaces are broad, shallow, and conformationally dynamic, optimized for extensive contact areas rather than high-affinity small-molecule binding [1]. Even in cases where pockets exist, such as the nucleotide-binding site of KRAS, they are typically highly conserved, transient, or difficult to access pharmacologically under physiological conditions [2–4]. Antibodies and biologics have extended therapeutic reach to extracellular targets; however, their large size, delivery constraints, and limited intracellular access restrict their applicability in many cancer-associated PPIs [5,6]. Consequently, new molecular modalities capable of engaging protein interfaces beyond the reach of antibodies and small molecules are highly desirable.

Among known PPI targets, the PD-1/PD-L1 immune checkpoint exemplifies an extracellular interface that has been successfully modulated therapeutically. Antibodies such as nivolumab, pembrolizumab, and others have demonstrated durable antitumor activity across cancer types, making immune checkpoint blockade a central component of oncology therapy [7–9]. Nevertheless, limitations remain, including off-target immune effects, large systemic exposure, cost, and challenges of tissue penetration. Resistance mechanisms, heterogeneity of response, and the need for combination regimens further highlight the unmet need [10,11]. In particular, antibodies target relatively large surfaces but may not optimally engage defined hotspot residues within the interface. Structural investigations of PD-1/PD-L1 and antibody complexes have revealed diverse blocking mechanisms, including steric overlap with binding surfaces and epitope tuning [12].

In contrast to PD-L1, MYC represents a nuclear PPI target that has eluded durable pharmacologic inhibition. Aberrant MYC signaling supports proliferation, metabolism alteration, immune evasion, and genome instability [13,14]. Yet direct targeting of MYC has long been considered undruggable, largely because MYC binds DNA and forms heterodimers (e.g., with MAX) via a structurally flexible, shallow interface lacking a clear small-molecule binding pocket [15,16]. Several small-molecule inhibitors, such as 10058-F4, can disrupt MYC/MAX dimerization, though they generally suffer from limited potency and pharmacokinetic stability [17]. The MYC miniprotein inhibitor Omomyc (OMO-103) has recently entered the clinic, demonstrating safety and early signs of clinical activity in solid tumors [18]. Despite these advances, the MYC/MAX interface remains a challenging and incompletely resolved therapeutic target, motivating alternative de novo design strategies capable of achieving precise, high-affinity engagement of this dynamic binding surface [15,16].

KRAS represents a cytoplasmic signaling node that, like MYC, was long viewed as intractable but has recently seen progress through allele-specific inhibitors. The advent of covalent small-molecule inhibitors targeting the KRAS G12C mutant (e.g., sotorasib, adagrasib) has shifted that paradigm, demonstrating that select mutant alleles can be drugged [19–21]. However, those inhibitors face significant resistance (both primary and acquired), limited allelic coverage, and challenges beyond G12C [20,22,23]. Strategies to stabilize inactive KRAS conformations, disrupt switch regions, or target other mutant alleles remain lacking, representing major gaps.

Recent advances in computational protein design now offer a path toward bridging these gaps. Breakthroughs in structure prediction, most notably AlphaFold2, have enabled near-experimental accuracy in modeling protein folds directly from sequence [24]. In parallel, generative modeling approaches, including diffusion-based backbone generation and sequence–structure co-design, are transforming de novo binder discovery [25,26]. Methods that flexibly sample backbone geometries and optimize sequences and conditions on interface constraints have expanded the exploration of the protein-interaction space, allowing the design of scaffolds specifically adapted to defined binding surfaces [27]. When integrated with energetic hotspot mapping, contact map analysis, and structure confidence evaluation, such pipelines can systematically yield compact, high-affinity candidates against historically intractable PPIs [28].

This study establishes and rigorously benchmarks a hotspot-guided de novo protein design pipeline centered on structural validation and physics-informed interface analysis. The work defines a reproducible framework for conditioning generative design on energetically dominant interface residues and evaluating structural self-consistency using force-field-based energetics and AlphaFold2 confidence metrics. This integrated infrastructure provides a scalable platform for targeting oncogenic protein–protein interfaces and lays the foundation for subsequent biochemical and functional evaluation.

In this work, we integrate BindCraft (v1.1.0) [29], a state-of-the-art generative binder design engine, with hotspot identification via MOE’s Protein Contacts module and structural validation via AlphaFold2. This in silico framework was applied to three representative oncogenic systems: the PD-1/PD-L1 immune checkpoint, the MYC/MAX transcriptional complex, and mutant KRAS. Across targets, the resulting designs exhibited high predicted structural confidence, thermodynamic stability, and consistent engagement of interfacial hotspots confirmed by contact map analysis. Collectively, these results demonstrate a generalizable computational pipeline that complements antibody- and small-molecule-based strategies, expanding the therapeutic landscape for previously intractable PPIs in immuno-oncology and oncogene-driven cancers.

## 2. Materials and Methods

### 2.1. Energetic Hotspot Mapping and Interface Analysis

Crystal structures of MYC/MAX (PDB 1NKP), PD-1/PD-L1 (PDB 5IUS), and KRAS/RAF (PDB 6XHB) were analyzed to identify energetic hotspots that define key binding determinants. Complexes were imported as biological assemblies, and chains were assigned as receptor and ligand for inter-chain interaction analysis. Residue–residue contact tables containing atomic distances and interaction energies were exported for downstream processing.

Energetic interactions were evaluated using either MOE Protein Contacts or the open DesignForge (v0.1.0) OpenMM-based implementation. In the latter, nonbonded electrostatic and Lennard–Jones interaction energies were computed using Amber14 force field parameters. Both approaches generated residue pair tables containing interatomic distances and total interaction energies for all inter-chain contacts.

### 2.2. Hotspot Definition and Selection Criteria

Hotspots were defined at the residue level by aggregating inter-chain atomic interactions within a 3.5 Å heavy-atom distance cutoff. A favorable contact was defined as a residue pair exhibiting a total nonbonded interaction energy ≤ −1.0 kcal mol^−1^ and at least one interatomic distance ≤ 3.5 Å. Interaction energies correspond to the summed electrostatic and van der Waals contributions.

Residue-level hotspot strength was calculated as the cumulative magnitude of favorable interaction energies for each residue. Residues were ranked by total favorable interaction energy contribution, and Top-N hotspot selections were used to condition backbone generation within the design workflow. Water molecules were excluded from contact aggregation. All energy thresholds and distance cutoffs were applied uniformly across complexes to ensure methodological consistency and comparability.

Energy-weighted histograms were generated to visualize dominant hotspot residues on both binding partners. Three complementary geometric representations, Cα–Cα distance matrices, reciprocal-distance (100/d) maps, and binary (≤8 Å) contact matrices, were constructed to visualize the spatial organization of the interface. These energetic and spatial features serve as inputs to downstream DesignForge modules.

### 2.3. Open DesignForge Hotspot Mapping

In addition to MOE-based hotspot extraction, energetic hotspot mapping was independently implemented within the DesignForge framework using an OpenMM-based physics engine. Structural coordinates were preprocessed using PDBFixer to standardize residue identities, replace nonstandard residues, add missing heavy atoms, and protonate structures at pH 7.0. Nonbonded inter-chain interaction energies were computed using the Amber14 ff14SB force field without nonbonded cutoff truncation. For each receptor–ligand atom pair within a 4.5 Å distance threshold, Lennard–Jones and electrostatic interaction components were calculated. Electrostatics were evaluated using either vacuum conditions or a screened, distance-dependent dielectric approximation; screened electrostatic energies were used for residue-level hotspot aggregation.

Per-residue hotspot strength was defined as the cumulative magnitude of favorable inter-chain interaction energies. Favorable contacts were defined as atom pairs with negative total nonbonded energy expressed in kcal mol^−1^. Residue-level scores were calculated as the sum of absolute favorable interaction magnitudes across all inter-chain contacts involving a given residue. Water molecules were excluded from aggregation. All geometric thresholds and scoring criteria were applied uniformly across complexes to ensure methodological consistency. Residues were ranked by total favorable interaction magnitude, and Top-N hotspot residues were used to condition backbone placement during downstream binder generation. The OpenMM-based module generates atom-level contact tables, energy-weighted hotspot summaries, and residue-level rankings for each complex.

### 2.4. Benchmarking of DesignForge Against MOE

To evaluate concordance between DesignForge and MOE hotspot identification, residue-level hotspot rankings were quantitatively compared across MYC/MAX (PDB 1NKP), PD-1/PD-L1 (PDB 5IUS), and KRAS/RAF (PDB 6XHB) complexes. MOE Protein Contacts output tables were exported as residue pair interaction matrices and aggregated to residue-level hotspot strengths using the same geometric and energetic criteria applied in the DesignForge workflow. Specifically, inter-chain contacts were filtered using identical distance thresholds, and favorable interactions were defined according to negative total nonbonded energy contributions.

Residue identifiers were canonicalized to a consistent chain residue format prior to comparison, and water residues were excluded from both datasets. Concordance between methods was assessed using Spearman rank correlation of residue rankings for shared hotspot residues and Pearson correlation of hotspot strength magnitudes. In addition, Top-N overlap analyses were performed at multiple thresholds (N = 5, 10, 20, 30, and 50 residues) to compute intersection size, Jaccard index, and recall relative to each method. These quantitative benchmarking procedures establish the degree to which the open DesignForge implementation reproduces energetic cores identified by MOE across structurally and functionally distinct oncogenic interfaces.

### 2.5. Binder Design Pipeline and Structural Validation

The design workflow integrated hotspot coordinates with BindCraft, a generative protein design platform that performs de novo binder construction using AlphaFold2-Multimer-guided hallucination and iterative backbone propagation. In this framework, candidate binder backbones are generated through cycles of structure hallucination constrained by interface geometry and predicted interaction metrics. ProteinMPNN is subsequently used for sequence optimization of the generated scaffolds, and AlphaFold2-Multimer metrics are used for structural validation and ranking of candidate designs. Within the DesignForge framework, BindCraft functions as the binder generation engine, while hotspot coordinates derived from MOE or the open DesignForge mapping module provide spatial constraints that guide binder placement at the target interface.

For each sampled backbone, sequence optimization was performed using ProteinMPNN, a graph neural network-based protein sequence design model that predicts amino acid identities conditioned on a fixed backbone structure. ProteinMPNN maximizes the conditional likelihood of residue identities given local structural geometry, thereby selecting sequences predicted to stabilize the designed fold and interface. Multiple sequence variants were generated per backbone.

The 100 highest-confidence designs, meeting predefined thresholds of mean pLDDT ≥ 80 and iPAE ≤ 15 Å^2^ following AlphaFold2-Multimer evaluation, were re tained for downstream analysis. Here, pLDDT (predicted local distance difference test) is the per-residue confidence score reported by AlphaFold on a 0–100 scale, with higher values indicating greater local structural reliability. iPAE (interface predicted aligned error) represents the predicted alignment error computed across inter-chain residue pairs at the binding interface, reflecting confidence in the relative orientation of binder and target chains.

Final models were rendered in PyMOL (v3.1.6.1) to assess binder orientation and structural convergence. Candidate binders were ranked using quantitative metrics including helicity, hotspot engagement counts, predicted interface residues, ProteinMPNN scores, and AlphaFold2-derived binder RMSDs. DesignForge further incorporates downstream evaluations such as energy–distance correlation plots, contact map visualization, and sequence-entropy profiling to support the systematic selection of high-quality designs.

All three complexes evaluated in this study were analyzed using both MOE Protein Contacts and the open DesignForge hotspot module and processed through the full pipeline. Although the PD-1/PD-L1 structural template was previously used as an illustrative example in the original BindCraft publication, all binder backbones, sequences, and downstream metrics generated here are new. The MYC/MAX and KRAS/RAF systems represent new applications of the pipeline.

### 2.6. Ensemble Sequence and Structural Analyses

Sequence characteristics across the ensemble were summarized using positional frequency logos derived from ProteinMPNN output alignments. Pairwise sequence identity matrices were computed to quantify convergence, and density clustering was used to identify low-iPAE, high-confidence subsets. Sequence embeddings were projected into three-dimensional space using uniform manifold approximation and projection (UMAP; k = 3) to visualize diversity across designs.

Electrostatic surface potentials for top binders were computed using Poisson–Boltzmann calculations within PyMOL and rendered on solvent-accessible surfaces to assess charge complementarity.

Ensemble-level metrics were derived from a unified design metrics file, a structured tabulated output generated for each candidate binder that consolidates structural confidence metrics (mean pLDDT, interface predicted aligned error, RMSD), interface-contact descriptors (predicted interface residues, hotspot engagement counts), and sequence-level statistics (length, helicity, ProteinMPNN score). This consolidated metrics table enables standardized ranking, threshold-based filtering, and cross-target comparison.

Scatterplots of iPAE versus pLDDT were used to evaluate structural confidence clustering. Pairwise sequence identity matrices revealed moderate similarity, indicating partial convergence toward common structural folds.

### 2.7. Structural Deviation Analysis

Backbone deviation of each designed binder relative to its native protein–protein interface was quantified using per-residue root-mean-square deviation (RMSD). For MYC designs, the top model was structurally aligned to the MYC chain from the MYC/MAX complex (PDB 1NKP). RMSD values were computed for backbone atoms (N, Cα, C, O) after least-squares superposition and plotted along the aligned residue index. Interface residues were defined based on the hotspot criteria described above and highlighted in downstream visualizations. Equivalent procedures were applied to PD-1/PD-L1 (PDB 5IUS) and KRAS/RAF (PDB 6XHB) complexes to generate per-residue RMSD profiles for cross-target comparison.

### 2.8. Interface Quality and Energy–Distance Correlation

Inter-residue interaction energies and centroid-to-centroid distances were extracted directly from the MOE Protein Contacts tables used for hotspot mapping. For each residue pair, the mean interatomic distance and total interaction energy (kcal mol^−1^) were plotted as a scatter distribution to assess packing quality. The Pearson correlation coefficient (r) from linear regression quantified the expected inverse relationship between contact distance and interaction energy. Correlation values were observed across designed interfaces, reflecting realistic side-chain packing and physically coherent nonbonded interactions.

### 2.9. Sequence Diversity Metrics

All designed binder sequences were aligned using pairwise Needleman–Wunsch global alignment implemented in Python 3.10.19.

Normalized Hamming distances were computed for every sequence pair to measure global sequence divergence. The resulting distribution histograms represent overall sequence diversity for the ensemble (Supplementary Figure S4D).

Positional sequence variability was quantified using Shannon entropy (*H*_s_) calculated from multiple sequence alignments according to

$$H_{s}=-\sum_{i}p_{i}{\text{log}}_{2}\;p_{i}$$

where *p*_*i*_ is the observed frequency of residue *i* at each alignment column. Entropy values were plotted as a function of residue position (Supplementary Figure S4D) to identify conserved and variable regions. Interface residues consistently showed minimal entropy, while loop and solvent-exposed regions displayed moderate variability.

### 2.10. Cross-Target Comparative Analyses

The same RMSD, energy distance, and sequence diversity pipelines were applied to the PD-L1 and KRAS binder ensembles, which are presented side-by-side in Supplementary Figures S3 and S4. Each figure includes results for both systems to enable direct visual comparison of structural and sequence-based metrics. For PD-L1 binders, alignments were performed to the PD-1 receptor chain using Cα superposition, whereas for KRAS binders, alignments were based on the switch I-proximal effector-binding surface of KRAS. All analyses used identical thresholds and plotting parameters to ensure comparability across target classes.

## 3. Results

### 3.1. Hotspot Mapping and Structural Characterization of Oncogenic Protein–Protein Interfaces

To delineate the structural and energetic features governing oncogenic protein–protein interactions, we first analyzed representative complexes of MYC-MAX (PDB 1NKP), PD-1/PD-L1 (PDB 5IUS), and KRAS/RAF (PDB 6XHB) using the Molecular Operating Environment (MOE) Protein Contacts module (Table S1). This approach quantifies all inter-residue contacts across each interface, assigning both geometric distance and energetic contribution to every residue pair. The resulting profiles highlight residue clusters that make dominant contributions to binding stability, interaction hotspots that define the structural framework for subsequent binder design.

**Figure 1.**
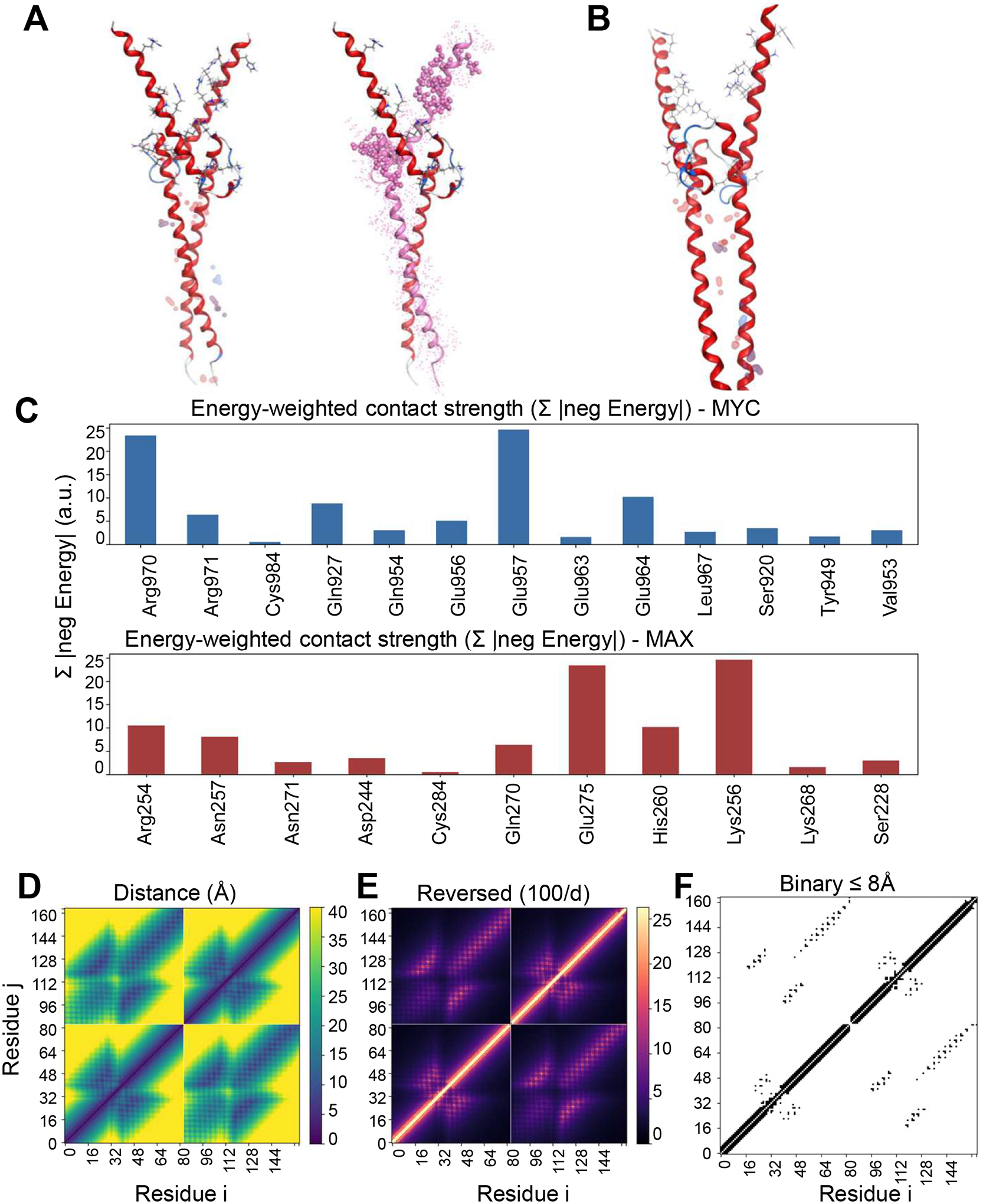
Hotspot mapping and contact analysis of oncogenic interfaces. (**A**) Crystal structure of the MYC-MAX complex (PDB 1NKP) with full heterodimer (left) and MYC highlighted (right). (**B**) MYC–MAX interface showing representative residue–residue contacts that mediate heterodimer formation. (**C**) Energy-weighted residue histograms for MYC (top) and MAX (bottom), where bar height represents the sum of absolute negative interaction energies (Σ|Eneg|, arbitrary units). (**D**–**F**) Contact maps derived from Cα coordinates of the MYC-MAX complex: (**D**) distance matrix (Å), (**E**) reciprocal-distance map (100/d), and (**F**) binary contact map showing residue pairs ≤ 8 Å. Compact interfacial clusters delineate residues mediating the MYC-MAX interaction.

In the MYC-MAX heterodimer, hotspot mapping (Figure 1A,B) highlighted a narrow strip of energetically dominant residues along the coiled–coil interface of the basic helix–loop–helix leucine-zipper region. These residues form a repeating pattern of hydrophobic and electrostatic contacts consistent with prior biochemical studies of MYC/MAX complex formation [30]. The three-dimensional view of the full heterodimer and isolated MYC chain (Figure 1A) emphasizes the exposed helical surface that constitutes the principal binding interface. The MOE energy map (Figure 1B) and residue-wise energy histograms (Table S1) revealed contiguous patches of favorable contacts centered on Gln927, Glu957, Glu964, Leu967, and Arg970 on MYC and on Arg254, Lys256, His260, Asn271, and Glu275 on MAX, delineating the core of the MYC–MAX dimerization interface. These hotspots cluster into two continuous bands along the MYC helix, indicating that most of the interaction energy is concentrated within a small subset of residues.

Energy-weighted contact histograms (Figure 1C) confirmed that the most stabilizing contributions arise from a small cluster of residues, namely, Glu957, Glu964, and Arg970 on MYC engaging Lys256, His260, and Glu275 on MAX. These strong electrostatic contacts account for most of the cumulative negative interaction energy, producing a steep, non-uniform distribution that indicates binding energy is concentrated within a few localized helical turns rather than spread evenly across the interface.

Analogous analyses of PD-1/PD-L1 and KRAS/RAF complexes revealed similarly compact energetic cores (Figure S1). In PD-1/PD-L1, the strongest interactions localized around the central FG and BC loops of PD-1, overlapping regions that correspond to known antibody epitopes such as those targeted by nivolumab and pembrolizumab. In KRAS/RAF, contact mapping revealed a dominant high-density cluster centered on the switch I region and adjacent β2-β3 loop of KRAS, which together form the canonical effector-binding surface for RAF. Although switch II undergoes conformational changes during nucleotide exchange, it does not directly engage the RAF interface in this complex. These results reinforce that even in large, seemingly diffuse protein interfaces, binding stability is governed by a restricted set of geometrically and energetically privileged residues.

For the PD-1/PD-L1 system, we also compared predicted binder quality metrics with those reported previously for PD-L1 binders [29], noting that both sets exhibit high structural confidence with pLDDT values greater than 0.90 and interface pAE values within the ~1–3 Å range that characterizes reliable predicted interfaces, despite sampling different binder length and sequence distributions.

To complement the energetic analyses, we computed Cα-Cα distance matrices, reciprocal-distance maps, and binary contact matrices using a custom chain-aware Python script (Figure 1D–F). The distance map (Figure 1D) revealed continuous, symmetric interaction bands corresponding to the principal dimerization interface, displaying the periodic lattice typical of parallel helices. The reciprocal-distance representation (Figure 1E) accentuated short-range contacts, separating the high-density interfacial core from peripheral residues. Finally, the binary contact map (Figure 1F) applied an 8 Å cutoff to delineate discrete contact regions, clearly outlining the contiguous inter-helical band that coincides with the energetic hotspots. Together, these geometric representations complement the MOE energy profiles and confirm the spatial organization of stabilizing contacts within the interface.

Collectively, these analyses establish a quantitative description of the molecular determinants that underlie three classes of therapeutically relevant PPIs, nuclear (MYC/MAX), extracellular (PD-1/PD-L1), and cytoplasmic (KRAS/RAF). Across all systems, hotspot residues form compact, energetically concentrated clusters that coincide with structurally accessible and solvent-exposed regions, making them ideal anchor points for de novo binder design. The integration of MOE-derived energy mapping with residue-level histograms and distance-based visualization provides a reproducible and generalizable framework for identifying interface features that can be leveraged in the computational generation of selective protein binders.

### 3.2. OpenMM-Based Hotspot Mapping and Benchmarking Against MOE

To ensure that hotspot identification within the DesignForge workflow is not dependent on proprietary software, we implemented an open-source hotspot mapping module based on OpenMM using the Amber14SB force field. This implementation computes explicit inter-chain nonbonded interactions, including Lennard–Jones and electrostatic contributions, at the atom level and aggregates them to residue-level hotspot strengths. Residues were ranked by the summed magnitude of favorable interaction energies under uniform geometric cutoffs and labeling conventions matched to those used in the MOE-based analysis. Water molecules were excluded from aggregation, and identical distance thresholds were applied across complexes to enable direct benchmarking.

We benchmarked the OpenMM-derived hotspot maps against MOE outputs for MYC/MAX (PDB 1NKP), PD-1/PD-L1 (PDB 5IUS), and KRAS/RAF (PDB 6XHB) (Figures S2–S4). Across all three complexes, the highest-ranked interfacial residues identified by MOE were consistently recovered by the OpenMM implementation. Normalized hotspot-strength profiles showed strong agreement among dominant residues (Figures S2A, S3A and S4A), and Top-N overlap analyses demonstrated substantial convergence within the Top-10 to Top-40 ranked positions (Figures S2B, S3B and S4B). Jaccard similarity and reciprocal recall curves indicated stable prioritization of a shared energetic core across increasing residue thresholds.

Rank comparisons among residues identified by both approaches demonstrated overall positive associations in relative ordering (Figures S2C, S3C and S4C), with the strongest concordance observed for the more compact interfaces. Residue-level hotspot-strength magnitudes also exhibited consistent positive trends despite differences in absolute energy scales arising from distinct scoring formulations (Figures S2D, S3D and S4D). Agreement was particularly robust for MYC/MAX and PD-1/PD-L1, which display concentrated energetic cores dominated by electrostatic and short-range packing interactions. For the broader KRAS/RAF interface, rank dispersion was moderately greater, consistent with its more distributed switch-region contact architecture; however, key effector-binding residues remained consistently prioritized by both methods.

Because DesignForge conditioning relies on relative hotspot ranking rather than absolute energy magnitude, the observed concordance in residue prioritization confirms that hotspot-guided binder conditioning is robust to implementation choice. These benchmarking results demonstrate that the energetic landscapes defining interfacial cores are reproducible across independent computational frameworks, validating the open-source DesignForge hotspot module as a consistent replacement for MOE-based mapping within the overall design pipeline.

### 3.3. Designed Binder Structures and Validation Results

**Figure 2.**
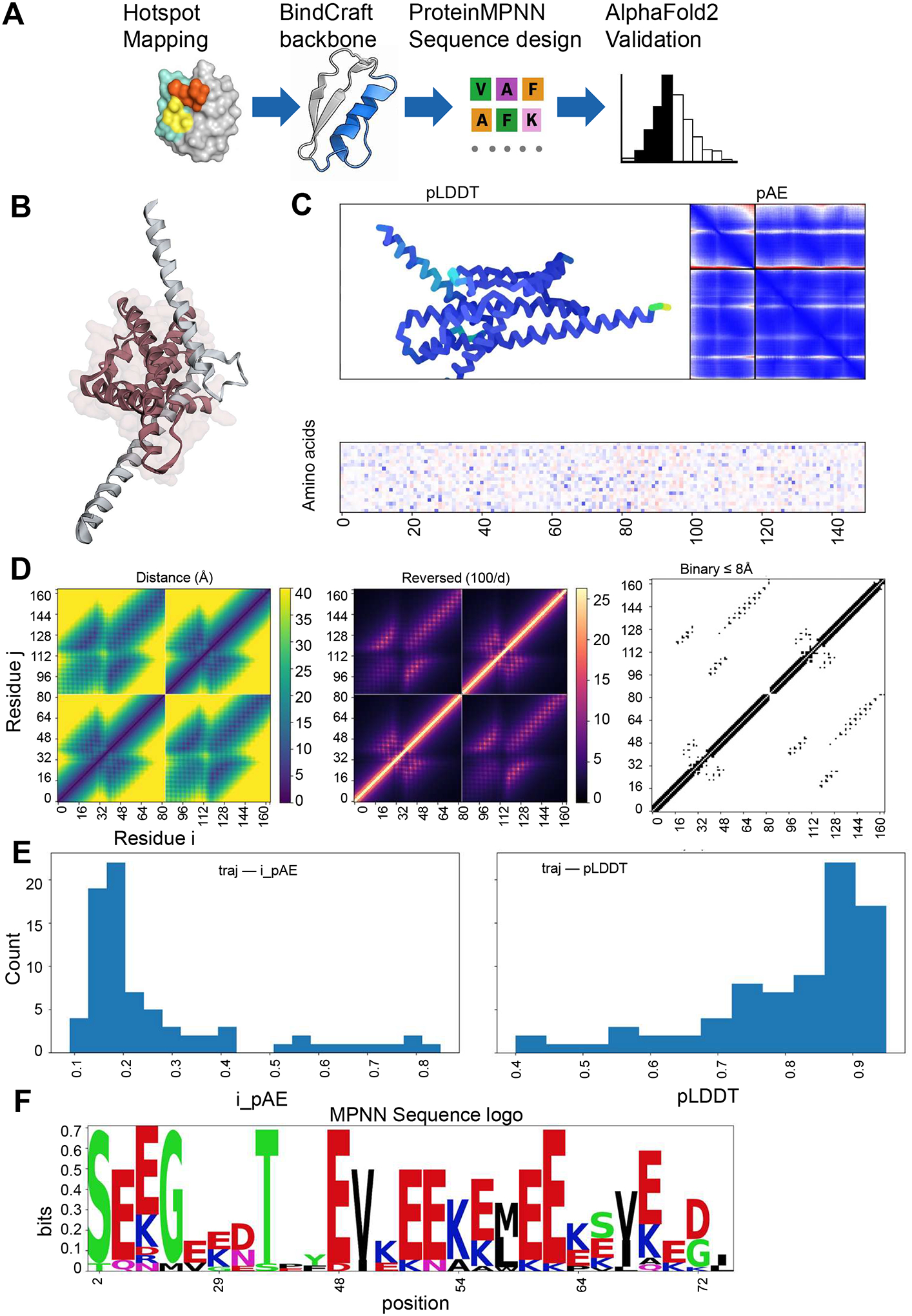
Binder design pipeline and model validation. (**A**) Schematic overview of the de novo binder design workflow: (1) hotspot analysis, (2) BindCraft scaffold generation, (3) RFDesign refinement, (4) AlphaFold2 structure validation. (**B**) Top-ranked MYC binder model superimposed on the native MYC helix from PDB 1NKP. (**C**) Representative animation frame from AlphaFold2 refinement trajectory showing stable binding orientation with low interface-predicted aligned error (iPAE). (**D**) Distance, reciprocal-distance, and binary (≤8 Å) contact maps confirming complementary shape and interface packing between MYC and binder. (**E**) iPAE and predicted local distance difference test (pLDDT) distributions, demonstrating high structural confidence and accurate backbone geometry. (**F**) Sequence-logo visualization (information content, bits) highlighting conserved hydrophobic and charged residues within the designed binding interface.

To extend the hotspot analyses toward binder generation, we used an integrated computational workflow that combines molecular interface mapping, guided backbone generation, sequence design, and structure-based evaluation. The pipeline (Figure 2A) links four sequential stages: hotspot identification, de novo backbone generation with BindCraft conditioned on hotspot geometry, sequence design with ProteinMPNN, and structural validation with AlphaFold2-Multimer. Together, these components form a complete in silico framework for creating compact, high-confidence protein binders targeting the MYC/MAX interface and related oncogenic protein–protein interactions.

A representative top-ranked binder model generated through this workflow is shown in Figure 2B. The designed miniprotein adopts a compact α-helical fold that aligns along the MYC target helix and mirrors the hotspot geometry identified previously. The interface displays favorable side-chain complementarity near Gln927, Glu957, Glu964, Leu967, and Arg970 on MYC, recapitulating the key energetic contacts observed in the native MYC-MAX complex. The predicted binding orientation positions the designed scaffold to engage both hydrophobic and charged residues across the central coiled–coil region, consistent with the structural requirements for selective MYC recognition. Quantitative summaries for the top five ranked binders for each target are provided in Table S2.

Trajectory frames from the design refinement process revealed progressive stabilization of the interface and a steady decrease in predicted error. Animation snapshots extracted from the trajectory (Figure 2C) showed that the binder retained a consistent orientation along the MYC helix throughout optimization, with a low interface predicted aligned error (iPAE) value. The representative frame shown exhibits tightly packed helical segments and minimal backbone deviation, indicating a stable, well-defined docking configuration with low structural uncertainty at the interface.

To further assess geometric complementarity, chain-aware distance and contact maps were computed from the final ensemble (Figure 2D). The left panel shows the raw Cα-Cα distance matrix, revealing continuous regions of close proximity between binder and target. The middle panel presents the reciprocal-distance representation (100/d), which enhances visualization of short-range contacts concentrated within the interfacial core. The right panel displays the binary contact map (≤8 Å), highlighting a sharply defined band of interacting residues corresponding to the primary MYC binding groove. These complementary visualizations confirm that the designed binder reproduces the contact density and orientation of the native interface while maintaining a compact, self-consistent internal topology.

Beyond geometric validation, we evaluated model confidence and sequence trends among the top-ranked binder designs. Histogram distributions of iPAE and per-residue pLDDT values (Figure 2E) indicated that most trajectories achieved low iPAE (<0.25) and high pLDDT (>0.8) scores, signifying high-confidence, well-folded structures. These metrics suggest that the predicted binder–target complexes are geometrically consistent with native-like packing and exhibit minimal uncertainty at the modeled interface.

Sequence variability across the design ensemble was visualized using an MPNN-derived sequence logo (Figure 2F). Strong positional conservation occurred within the central helical region, dominated by hydrophobic (L, F, V) and charged (K, E) residues that mirror the physicochemical pattern of the MYC interface. Peripheral positions displayed greater diversity, reflecting tolerated variability outside the energetic core. The enrichment of leucine and lysine near positions corresponding to Glu957, Glu964, Leu967, and Arg970 supports the residue preferences inferred from the MOE energy mapping and contact map analyses.

Comparable design-stage analyses for PD-L1 and KRAS binder ensembles are shown in Supplementary Figure S5. These models recapitulate the key structural characteristics observed in the MYC designs, including compact α-helical scaffolds aligned along their respective target interfaces (Figure S5A), low predicted interface error and high per-residue confidence (Figure S5B), and dense interfacial contact bands revealed by distance, reciprocal-distance, and binary contact maps (Figure S5C). Histogram distributions of iPAE and pLDDT values (Figure S5D) confirm uniformly high model confidence, while sequence-logo analyses (Figure S5E) highlight strong positional conservation of hydrophobic and charged residues at hotspot-proximal sites. Together, these data demonstrate that the DesignForge workflow yields reproducible, geometrically consistent binders across multiple oncogenic targets. A full list of abbreviations and scoring terms used throughout this study is provided in Supplementary Table S3.

### 3.4. Computational Environment and Runtime

All binder designs in this study were generated using the Google Colab environment equipped with an NVIDIA A100-SXM4–40GB GPU. Installation of BindCraft and its dependencies required approximately two minutes. Individual generative runs typically executed within several minutes per backbone. Minimal examples can be executed on a standard CPU-only desktop; however, full DesignForge workflows are more efficiently run on GPU-enabled systems due to the computational cost of diffusion-based backbone generation and AlphaFold2 structural evaluation.

### 3.5. Electrostatic Surface Properties, Sequence Diversity, and Structural Confidence of Designed Binders

**Figure 3.**
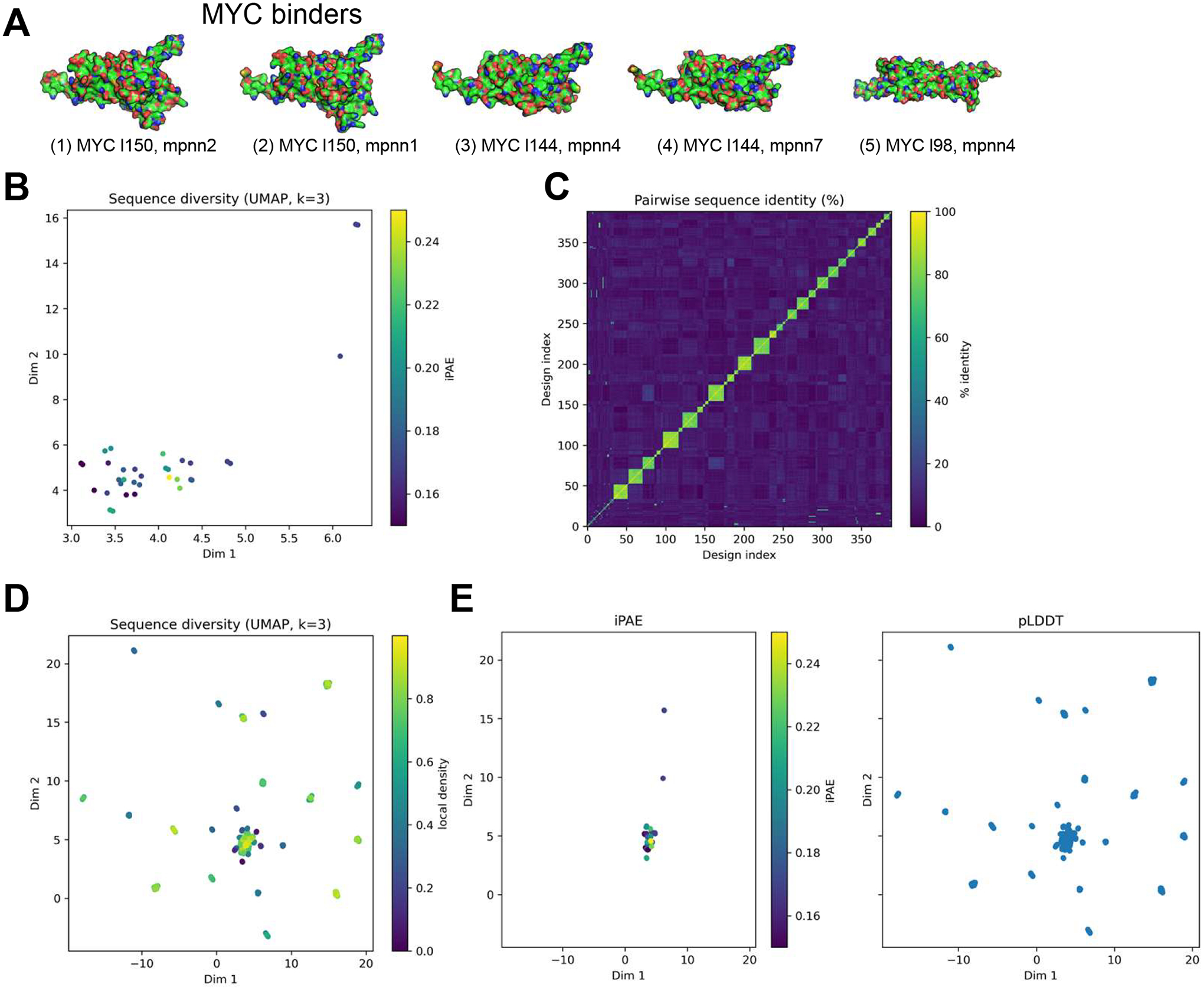
Electrostatics, sequence diversity, and structural metrics of MYC binders. (**A**) Electrostatic surface potential of the top MYC binders (blue = positive, red = negative, with intermediate colors representing a continuous gradient of electrostatic potential) showing charge complementarity with the MYC/MAX interface. (**B**,**D**) UMAP embeddings of binder sequence diversity colored by (**B**) iPAE and (**D**) pairwise sequence identity, generated from the design metrics CSV. (**C**) Pairwise sequence identity heatmap across top-ranked binders showing strong convergence within the design ensemble. (**E**) Scatterplots comparing iPAE and pLDDT scores for each binder, confirming high-confidence folding and consistent interface predictions.

Electrostatic potential mapping of the top binder models (Figure 3A) revealed distinct regions of positive and negative surface charge arranged in a pattern complementary to the MYC interface. The interfacial face exhibited a mosaic of alternating acidic and basic patches that align with the charged residues on MYC, suggesting favorable electrostatic complementarity at the binding surface. Across multiple orientations, the designed binder maintained a coherent charge distribution consistent with its intended role as a stabilizing partner to the largely hydrophobic and polar MYC helix.

To assess sequence diversity and structural convergence across the designed ensemble, we projected all variants into a low-dimensional sequence similarity space using uniform manifold approximation and projection (UMAP). The resulting embedding (Figure 3B) showed that most designs cluster within a compact region, consistent with convergence toward a shared backbone architecture despite underlying sequence variability. A few peripheral points represent more divergent sequences that nevertheless retain low predicted interface error.

Pairwise sequence identity analysis (Figure 3C) further confirmed substantial sequence diversity, with most designs sharing less than 50% identity. The prominent diagonal reflects unique sequences for nearly all designs, while only small local clusters exhibited moderate similarity. Together, these results indicate that the design ensemble spans a broad and heterogeneous sequence landscape while maintaining a consistent structural fold.

When the analysis was restricted to high-confidence models, the UMAP distribution (Figure 3D) revealed a dense central cluster containing the lowest-iPAE binders, surrounded by a broader set of moderately dispersed variants. This pattern indicates that a subset of geometrically consistent, structurally reliable designs emerged from a wider exploration of sequence space.

Relationships between predicted interface accuracy and model confidence were further visualized using iPAE and pLDDT projections (Figure 3E). Most designs clustered at low iPAE and high pLDDT values, underscoring the overall structural reliability of the ensemble. A few outliers exhibited higher error or reduced confidence, while the majority showed stable, well-converged interface geometries.

Collectively, these analyses show that the final MYC binder designs converge toward a shared structural architecture characterized by stable electrostatic complementarity, constrained sequence variability, and high predicted confidence, reflecting both the reproducibility and precision of the DesignForge pipeline.

### 3.6. Structural Deviation, Interface Quality, and Sequence Diversity Among Designed Binders

To evaluate structural fidelity and sequence convergence of the designed binders, we compared each model to its respective native protein–protein interface and analyzed correlations between backbone geometry, interfacial energetics, and sequence variability. These assessments quantify how accurately the computational designs recapitulate the physical and sequence constraints of their natural counterparts.

**Figure 4.**
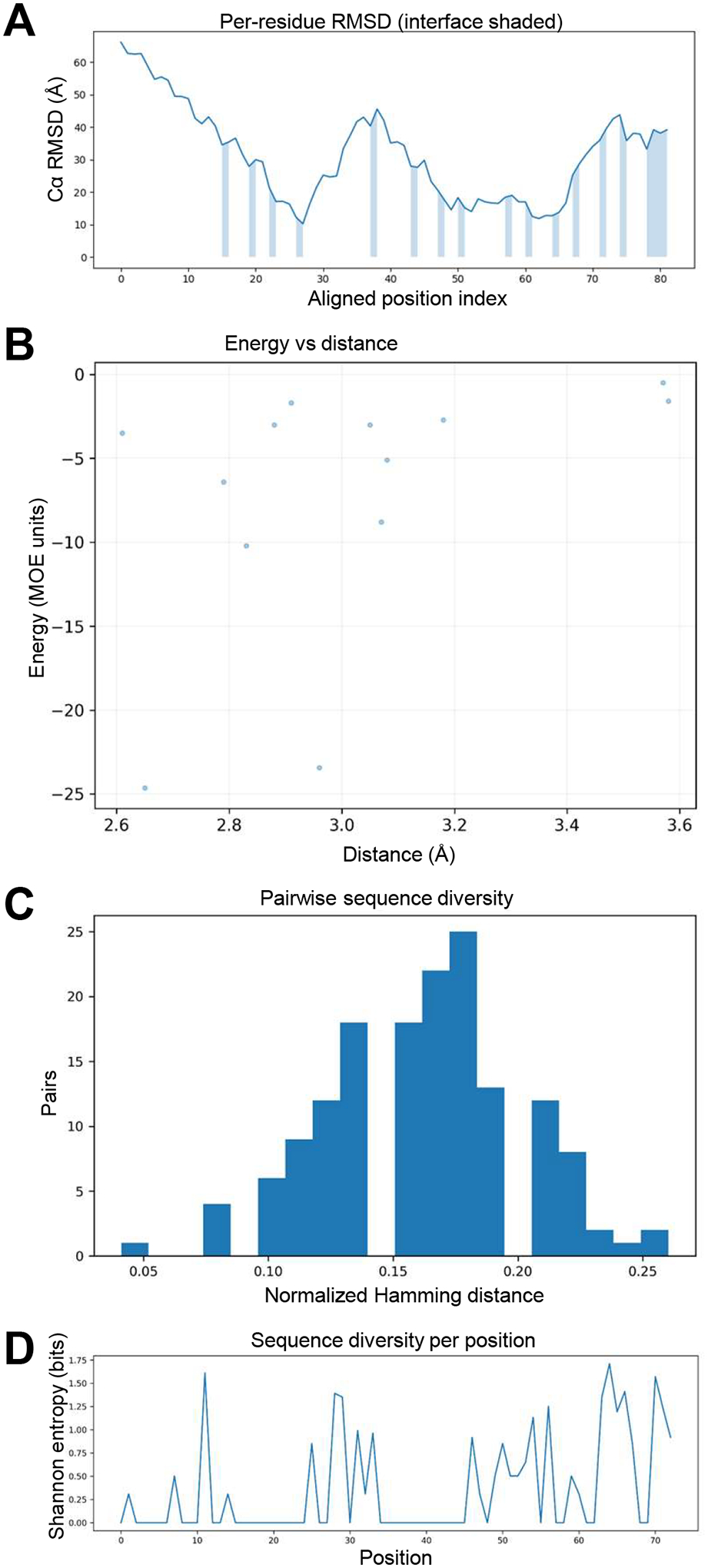
Structural deviation, interface quality, and sequence diversity among designed MYC binders. (**A**) Per-residue backbone RMSD between the top binder mode and the MYC chain from the MYC/MAX crystal structure (PDB 1NKP). The shaded band indicates the interface region; lower RMSD values within this region denote strong structural agreement with the native motif. (**B**) Scatterplot of interaction energy versus inter-residue distance. (**C**) Histogram of normalized Hamming distances illustrating sequence convergence among designed binders. (**D**) Shannon entropy per residue position, with low-entropy peaks corresponding to conserved interface residues and higher variability at solvent-exposed positions.

Per-residue RMSD analysis (Figure 4A) comparing the top MYC binder to the native MYC interface from the MYC/MAX complex showed close backbone alignment across the helical contact region, with deviations generally within 2–3 Å over most interfacial residues. The global interface RMSD of ~2.8 Å indicates strong structural fidelity to the target geometry. Local increases near the N- and C-terminal ends correspond to solvent-exposed regions outside the binding interface. The shaded segment denotes the interfacial zone, where the lowest RMSD values coincide with residues contributing the strongest energetic stabilization.

The relationship between inter-residue interaction energy and contact distance (Figure 4B) showed a moderate inverse correlation, consistent with residues in closer spatial proximity forming more favorable interactions. This trend reflects physically realistic side-chain packing and effective optimization of nonbonded contacts within the designed interface.

Sequence space analysis demonstrated that the ensemble of designed MYC binders converged toward a narrow set of related variants. The histogram of normalized Hamming distances (Figure 4C) showed a tight distribution consistent with modest sequence diversification around a dominant motif. This limited variability suggests selective optimization toward a stable fold while preserving a small degree of flexibility for local sequence adaptation.

Entropy mapping across aligned sequences (Figure 4D) delineated position-specific variability within the design ensemble. Peaks in Shannon entropy corresponded to loop or solvent-exposed regions, whereas interfacial residues showed minimal entropy, indicating strong conservation driven by structural and energetic constraints. These conserved positions coincide with the MYC hotspot residues identified by MOE contact analysis. These findings reinforce that sequence convergence reflects the physical determinants of binding stability.

To determine whether these design principles generalized across different target systems, we applied the same electrostatic, structural, and sequence diversity analyses to the PD-1/PD-L1 and KRAS/RAF binder ensembles (Supplementary Figures S6 and S7).

For PD-1/PD-L1, Coulombic electrostatic potential maps (Figure S6A,B) revealed contiguous patches of complementary positive and negative charge across the binder–PD–L1 interface, supporting favorable electrostatic complementarity. UMAP embeddings of the design library (Figure S6C,E) showed clustering of low-iPAE, high-confidence binders, consistent with structural convergence toward compact, reproducible folds. Pairwise sequence identity matrices (Figure S6D) indicated moderate overall sequence similarity, consistent with limited diversity across the PD-1 binder ensemble. When considered together with positional conservation patterns from sequence-logo analyses (Figure S5E), these results suggest that most sequence variability occurs outside the core interface, reflecting focused optimization around the PD-1 epitope.

For PD-L1 and KRAS binders, per-residue RMSD profiles (Figure S7A) revealed broader structural variation relative to their native interface templates, with global interface RMSDs of approximately 22 and 24 Å, respectively. Despite these large overall deviations, both profiles displayed periodic low-RMSD regions within the interface-shaded zones, indicating partial geometric correspondence along the target-contacting helices. The oscillating RMSD patterns suggest that while the designed binders adopt distinct global folds, they maintain localized structural alignment at interfacial residues critical for binding.

The energy–distance relationships (Figure S7B) showed weak or negligible correlations, indicating that favorable interaction energies occur over a broad range of contact distances rather than following a strict inverse trend. Sequence diversity analyses (Figure S7C,D) revealed substantial variability across the ensembles, with uniformly high positional entropy and broad pairwise Hamming-distance distributions. These results suggest that while the designed binders share a consistent overall topology, they explore a wide range of sequence solutions compatible with the target interface geometry.

The energy–distance relationship (Figure S7B) demonstrated that residues in closer proximity form more favorable interactions. Sequence diversity metrics (Figure S7C,D) revealed moderate variation across the ensemble with low positional entropy at interface-defining residues, consistent with strong conservation within the switch-region core.

Collectively, these cross-target analyses show that the designed binders for MYC/MAX, PD-1/PD-L1, and KRAS/RAF converge toward geometrically consistent, electrostatically complementary scaffolds characterized by reproducible folding, stable interfacial packing, and high predicted confidence.

## 4. Discussion

This work demonstrates a generalizable, hotspot-guided strategy for de novo miniprotein binder design that spans three mechanistically distinct PPIs: the nuclear MYC/MAX dimer, the extracellular PD-1/PD-L1 checkpoint pair, and the cytoplasmic KRAS/RAF signaling node. Using DesignForge, our integrated pipeline combining MOE-based energetic profiling, RFdiffusion backbone generation, ProteinMPNN sequence design, and AlphaFold2 validation produced compact scaffolds that (i) recapitulate near-native backbone geometry at interfacial residues, (ii) exhibit physically coherent energy-distance relationships, and (iii) converge to low-entropy sequence solutions at key hotspot positions. This pattern of sequence convergence likely arises from intrinsic structural constraints, specifically, the limited number of residue combinations compatible with the dense packing and electrostatic complementarity characteristic of high-affinity interfaces, rather than from restricted sampling by the design algorithm. Although our demonstration focused on three cancer-related interaction surfaces, the workflow itself is disease-agnostic and can be applied to any structured PPI. This generalizability reflects the reliance on energetic hotspot mapping rather than cancer-specific biology.

DesignForge builds directly on recent advances in generative and predictive protein modeling. RFdiffusion generates backbones that satisfy functional constraints while sampling diverse topologies, supporting binder, oligomer, and motif-scaffolding applications within a unified framework [25]. ProteinMPNN then selects sequences predicted to encode those backbones with high fidelity [31], improving foldability across both native and de novo structures. AlphaFold2 provides structure confidence metrics (pLDDT, PAE/iPAE) that correlate with experimental success across multiple design studies [24,32]. The hotspot-driven design strategy extends deep-learning-based functional-site scaffolding approaches, embedding key interfacial residues within compact, energetically optimized folds [33]. Together, these components convert residue-level energetic maps into compact, testable protein scaffolds.

In addition to incorporating BindCraft as the backbone generation module, Design-Forge extends prior workflows by integrating MOE-based energetic hotspot mapping, multi-stage structural filtering, and automated downstream metrics that are not provided in existing pipelines. These additions include energy–distance correlation analyses, contact map visualization, sequence-entropy profiles, and AlphaFold2 batch validation, which together provide quantifiable measures of interface quality and design convergence. This broader analytical framework differentiates DesignForge from BindCraft alone and supplies interpretable diagnostics for selecting candidates for future experimental assessment.

MYC/MAX: MYC remains a high-value but historically undruggable target. The clinical debut of the Omomyc-derived miniprotein (OMO-103) showed first-in-human feasibility for MYC/MAX disruption [18]. Our de novo binders, which recapitulate the MYC/MAX helical-zipper geometry and concentrate conservation at MOE-defined hotspots, align with the emerging view that compact miniproteins can effectively engage transcription-factor PPIs while maintaining favorable biophysical properties. Whereas OMO-103 is MYC-derived, our designs originate from distinct scaffolds that provide an orthogonal starting point for optimizing specificity, stability, and, potentially, immunogenicity profiles.

PD-1/PD-L1: Structural and biophysical studies have mapped the PD-1/PD-L1 interface and defined the epitopes targeted by therapeutic PD-1 antibodies such as pembrolizumab and nivolumab [34,35]. Multiple analyses highlight PD-1 loop regions, particularly FG and BC, as dominant antibody-binding hotspots, consistent with our ensemble results showing low RMSD and high conservation adjacent to these motifs [36–38]. The observed electrostatic complementarity and sequence convergence at loop-proximal positions mirror key features of productive PD-1/PD-L1 blockade interfaces.

KRAS/RAF: The KRAS-RAF interaction is mediated primarily by the RAF Ras-binding domain (RBD) engaging the switch I region and adjacent β2-β3 loop of KRAS. Structural studies (e.g., PDB 6XHB and related studies [39,40]) have shown how these extended contact surfaces stabilize signaling assemblies. In our models, per-residue RMSD profiles (Figure S4A) indicated large overall deviations relative to the native complex, yet with localized low-RMSD segments along the switch I-proximal effector-binding surface that preserve its geometric alignment. Energy–distance plots (Figure S4B) reveal weak or negligible correlations, suggesting that favorable contacts occur over a range of spatial separations rather than strictly following an inverse energy–distance trend. Sequence diversity analyses (Figure S4C,D) showed broad Hamming-distance distributions and moderate-to-high positional entropy, reflecting extensive sequence exploration across the ensemble. Collectively, these results suggest that while the designed KRAS binders adopt distinct global folds, they retain local interface features consistent with the engagement of the switch-region surface.

Three quantitative metrics underpin confidence in our designed binders: (i) structural proximity, reflected by low per-residue and interface RMSD values; (ii) physicochemical realism, evidenced by the expected inverse relationship between inter-residue energy and distance, indicating well-packed side chains; and (iii) sequence convergence, characterized by low positional entropy and narrow Hamming-distance distributions that capture reproducible folding constraints. Collectively, these features align with key predictors of success observed in recent experimental validations of RFdiffusion- and ProteinMPNN-based designs [25,31].

Two key conclusions emerge from this study. First, hotspot-aware generative design generalizes across structurally and chemically distinct protein–protein interfaces, yielding compact scaffolds whose electrostatics complement the target surface and whose sequence landscapes converge at functionally critical residues. Second, DesignForge introduces quantitative, human-interpretable diagnostics, including RMSD, energy–distance relationships, and sequence-entropy metrics, that enable the rational triage of computational candidates before experimental screening and provide explicit parameters for iterative refinement.

In the case of MYC/MAX, where clinical activity has now been demonstrated for a miniprotein class (OMO-103 [18]), our results suggest that de novo scaffolds could complement or even surpass derivative constructs through enhanced specificity and stability. For PD-1/PD-L1 and KRAS/RAF, where structural determinants and loop or switch flexibility are well characterized [34,35,39], the observed interface alignment and conservation patterns mirror known contact topologies and motivate targeted experimental validation.

### Computational Scope and Future Integration

While the present study focuses on computational design and structural validation, the results establish a rigorous and internally consistent framework for hotspot-conditioned de novo binder generation. Structural confidence metrics, including pLDDT, iPAE, and per-residue RMSD, demonstrate geometric plausibility and interface coherence across three mechanistically distinct protein–protein interfaces. Energy–distance relationships and sequence convergence analyses further support the physicochemical realism and reproducibility of interface engagement. These metrics collectively define a structured, multi-parameter validation strategy for computational binder triage prior to experimental screening.

Importantly, the benchmarking analyses demonstrate that the open DesignForge hotspot mapping module quantitatively reproduces energetic cores identified by MOE across diverse oncogenic complexes. This concordance establishes the methodological reliability of the physics-based residue-ranking framework and validates the energetic priors used to condition generative backbone placement. The agreement between independent implementations strengthens confidence that the observed hotspot patterns reflect underlying interface determinants rather than software-specific artifacts.

The present work therefore establishes a reproducible computational infrastructure that enables systematic, hotspot-aware exploration of challenging protein–protein interfaces. Experimental characterization, including affinity measurements and competition assays, represents the natural next step for translating these structurally coherent designs into functional binders. By providing an open, physics-informed pipeline with integrated benchmarking and diagnostic metrics, DesignForge reduces the search space and increases the interpretability of candidate selection prior to empirical testing.

## 5. Conclusions

Integrating physics-based hotspot mapping with generative protein design enables the creation of binder ensembles that converge on sequence-constrained solutions across three representative oncogenic protein–protein interfaces. By anchoring designs at densely interacting energetic cores and validating structural plausibility through AlphaFold-derived confidence metrics, this strategy provides a reproducible and interpretable blueprint for in silico binder generation. The incorporation of an open, OpenMM-based hotspot detection module, quantitatively benchmarked against MOE across multiple complexes, further establishes DesignForge as a scalable and scriptable alternative to proprietary workflows for energetic interface mapping.

This study focuses on computational design, structural validation, and methodological benchmarking as a foundation for systematic binder discovery. Experimental characterization of prioritized candidates, including affinity measurements and competition assays, represents the natural next phase for translating these structurally coherent designs into functional binders.

## Supplementary Material

SUPPLEMENTARY INFORMATION

Table S1

Table S2

Table S3

**Supplementary Materials:** The following supporting information can be downloaded at https://www.mdpi.com/article/10.3390/biologics6010009/s1, Figure S1: Comparative hotspot and contact-map analyses of PD-1/PD-L1 and KRAS/RAF complexes; Figure S2: Benchmark comparison of MOE and DesignForge hotspot mapping for PD-1/PD-L1 (PDB 5IUS); Figure S3: Benchmark comparison of MOE and DesignForge hotspot mapping for MYC/MAX (PDB 1NKP); Figure S4: Benchmark comparison of MOE and DesignForge hotspot mapping for KRAS/RAF (PDB 6XHB); Figure S5: Structural, contact-map, and sequence analyses of designed PD-L1 and KRAS binders; Figure S6: Electrostatic, structural, and sequence-level analyses for PD-1/PD-L1 binders; Figure S7: Structural deviation, energetic correlation, and sequence-diversity analyses for KRAS/RAF binders. Table S1: Inter-residue contact profiles of representative oncogenic protein–protein complexes analyzed using MOE. Table S2. Top five binders for each protein-protein interaction. Table S3. Abbreviations used in the manuscript.

## Data Availability

All custom scripts used for contact map generation, binder design, and figure production are openly available at https://github.com/KidderLab/DesignForge (accessed on 15 March 2026).
