# Supplementary material for: De Novo Protein Design Enables Targeting of Intractable Oncogenic Protein–Protein Interfaces
Source: Biologics (Basel). Author manuscript; Available in PMC 2026 May 30. (PMC13221196; doi:10.3390/biologics6010009)
Supplement: SUPPLEMENTARY INFORMATION [file NIHMS2176950-supplement-SUPPLEMENTARY_INFORMATION.pdf]

**SUPPLEMENTARY INFORMATION**

# De novo protein design enables targeting of intractable oncogenic protein-protein interfaces

Varshika Ram Prakash[1-2], Yusuf Najy[1-2], Kalel Garrett[1-2], Brian F.P. Edwards[3], Benjamin L. Kidder[1-2*]

[1]Department of Oncology, Wayne State University School of Medicine, Detroit, MI, USA

[2]Karmanos Cancer Institute, Wayne State University School of Medicine, Detroit, MI, USA

[3]Department of Biochemistry, Microbiology and Immunology, Wayne State University, Detroit, MI, USA.

Running title: De novo designed binders for intractable oncogenic interactions.

[*]Correspondence:

Benjamin L. Kidder

Email : benjamin.kidder@wayne.edu

## SUPPLEMENTAL FIGURES

**Figure S1. Comparative hotspot and contact-map analyses of PD-1/PD-L1 and KRAS/RAF complexes.**

**(A-C)** Energy-weighted residue histograms from MOE interaction tables for PD-1 versus PD-L1 (**A**) and KRAS versus RAF (**C**). Bar height reflects the summed magnitude of negative interaction energies (Σ|Eneg|, arbitrary units), highlighting residues that contribute most strongly to interface stabilization. **(B)** Structural mapping of PD-1 energetic hotspots onto the PD-1/PD-L1 complex, showing clustering at the BC and FG loops. **(D-F)** PD-1/PD-L1 inter-chain contact maps computed from Cα coordinates: **(D)** distance matrix (Å), **(E)** reversed-distance representation (100/d), and **(F)** binary contact map (≤ 8 Å). The continuous high-density band corresponds to PD-1 BC and FG loops engaging PD-L1. **(G–I)** KRAS/RAF contact maps generated with identical parameters: **(G)** distance, **(H)** reversed-distance, and **(I)** binary (≤ 8 Å). Compact contact clusters align with the KRAS switch I and β2–β3 elements that interact with the RAF Ras-binding domain.

**Figure S2. Benchmark comparison of MOE and DesignForge hotspot mapping for PD-1/PD-L1 (PDB 5IUS).**

(A) Normalized hotspot strengths for top-ranked residues identified by MOE and the OpenMM-based DesignForge implementation. Residue-level hotspot strength was calculated as the summed magnitude of favorable inter-chain interaction energies. Values were normalized independently for each method to the maximum hotspot strength for visualization. (B) Top-N overlap analysis comparing residue rankings between MOE and DesignForge. Jaccard similarity, recall relative to MOE, and recall relative to DesignForge

are shown as a function of increasing residue cutoff (Top-N). (C) Rank comparison of shared hotspot residues identified by both methods. Each point represents a residue present in both ranked lists. Lower rank values indicate stronger hotspot contribution. (D) Residue-level hotspot strength comparison for residues identified by both methods. Strength corresponds to the sum of favorable interaction magnitudes per residue.

**Figure S3. Benchmark comparison of MOE and DesignForge hotspot mapping for MYC/MAX (PDB 1NKP).**

(A) Normalized hotspot strengths for top-ranked residues derived from MOE and the OpenMM-based DesignForge implementation. Residue-level strengths were calculated using summed favorable interaction magnitudes and normalized within each method. (B) Top-N overlap analysis showing Jaccard similarity and reciprocal recall metrics across increasing residue thresholds. (C) Rank–rank comparison of shared hotspot residues between methods. Lower rank values correspond to higher hotspot priority. (D) Comparison of residue-level hotspot strengths (sum of favorable interaction magnitudes) for residues present in both ranked sets.

**Figure S4. Benchmark comparison of MOE and DesignForge hotspot mapping for KRAS/RAF (PDB 6XHB).**

(A) Normalized hotspot strength profiles for top-ranked residues identified by MOE and the OpenMM-based DesignForge implementation. Strength values were independently normalized for each method. (B) Top-N overlap analysis displaying Jaccard similarity and recall metrics as a function of residue cutoff. (C) Rank comparison for residues shared

between MOE and DesignForge hotspot rankings. (D) Correlation of residue-level hotspot strengths between methods for shared residues.

**Figure S5. Structural, contact-map, and sequence analyses of designed PD-L1 and KRAS binders.**

**(A)** Ribbon representations of the top-ranked PD-L1 and KRAS binder designs, each adopting a compact α-helical scaffold positioned along its respective target interface. **(B)** Representative AlphaFold2 structure predictions for PD-L1 and KRAS binders with per-residue interface-predicted aligned error (iPAE) plots, showing uniformly low error and stable backbone geometry across refinement frames. **(C)** Chain-aware contact maps for both binder complexes: *top*, PD-L1 binder; *bottom*, KRAS binder. Each set shows distance (left), reciprocal-distance (100/d, middle), and binary (≤ 8 Å, right) representations. Both designs exhibit dense interfacial contact bands consistent with native binding topologies. **(D)** Histogram distributions of iPAE and predicted local distance difference test (pLDDT) values across AlphaFold2 trajectories: *top*, PD-L1 binder; *bottom*, KRAS binder. Both profiles indicate high structural confidence and minimal predicted interface error. **(E)** Sequence logos derived from top-ranked ProteinMPNN designs: *top*, PD-L1 binder; *bottom*, KRAS binder. Conserved hydrophobic and charged residues dominate buried interfacial positions.

**Figure S6. Electrostatic, structural, and sequence-level analyses for PD-1/PD-L1 binders.**

**(A)** Electrostatic surface potential of the top PD-L1 binder model (*blue = positive; red = negative*), showing complementary charge distribution relative to the PD-1 interface. **(B)** Electrostatic surface potential of the top KRAS binder model, illustrating charge complementarity and interfacial polarity aligned with the RAF-binding surface. **(C)** Pairwise sequence-identity heatmaps for the PD-L1 (left) and KRAS (right) binder ensembles. **(D)** Uniform Manifold Approximation and Projection (UMAP) embeddings of the complete PD-L1 (left) and KRAS (right) binder libraries, where each point represents a designed sequence colored by iPAE. Low-iPAE, high-confidence designs cluster tightly, indicating reproducible backbone geometries and consistent interface solutions across independent trajectories. **(E)** Scatterplots of iPAE versus pLDDT for PD-L1 (left) and KRAS (right) binders, demonstrating dense clustering in the high-confidence/low-error regime and confirming robust structural reliability of the top-ranked designs.

**Figure S7. Structural deviation, energetic correlation, and sequence-diversity analyses for KRAS/RAF binders.**

**(A)** Per-residue backbone root-mean-square deviation (RMSD) of the top PD-L1 (left) and KRAS (right) binders relative to their native complexes (PD-1/PD-L1, PDB 5IUS; KRAS/RAF, PDB 6XHB). **(B)** Correlation between inter-residue interaction energy and spatial distance across interface pairs for PD-L1 (left) and KRAS (right) binders. **(C)** Histograms of normalized Hamming distances within each binder. **(D)** Per-residue Shannon entropy profiles.

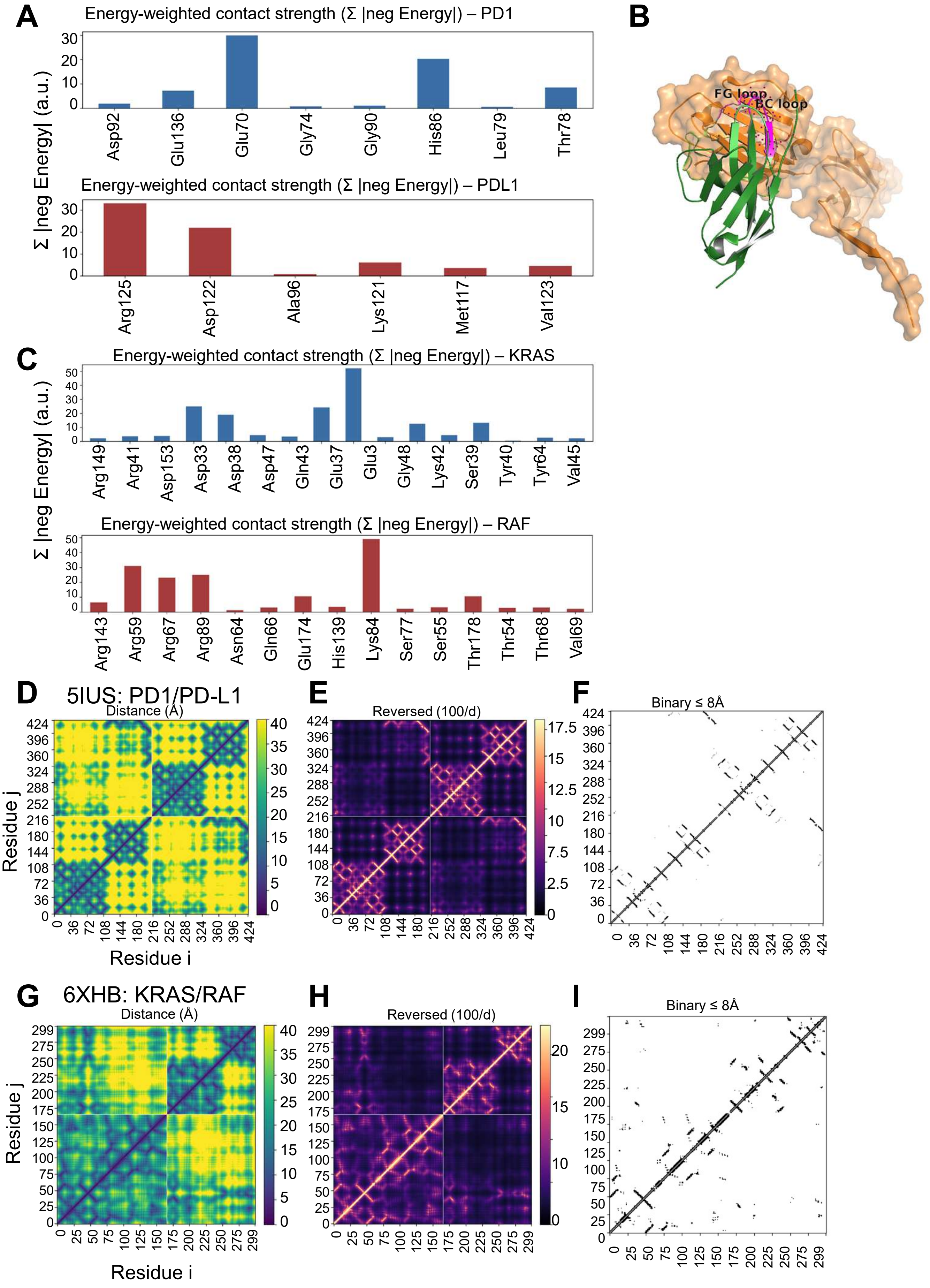

A
Energy-weighted contact strength (Σ |neg Energy|) – PD1
Asp92
Glu136
Glu70
Gly74
Gly90
His86
Leu79
Thr78
Energy-weighted contact strength (Σ |neg Energy|) – PDL1
Arg125
Asp122
Ala96
Lys121
Met117
Val123
Σ |neg Energy| (a.u.)
B
FG loop
BC loop
C
Energy-weighted contact strength (Σ |neg Energy|) – KRAS
Arg149
Arg41
Asp153
Asp33
Asp38
Asp47
Gln43
Glu37
Glu3
Gly48
Lys42
Ser39
Tyr40
Tyr64
Val45
Energy-weighted contact strength (Σ |neg Energy|) – RAF
Arg143
Arg59
Arg67
Arg89
Asn64
Gln66
Glu174
His139
Lys84
Ser77
Ser55
Thr178
Thr54
Thr68
Val69
Σ |neg Energy| (a.u.)
D
5IUS: PD1/PD-L1
Distance (Å)
Residue j
Residue i
E
Reversed (100/d)
F
Binary ≤ 8Å
G
6XHB: KRAS/RAF
Distance (Å)
Residue j
Residue i
H
Reversed (100/d)
I
Binary ≤ 8Å


**Figure S1**

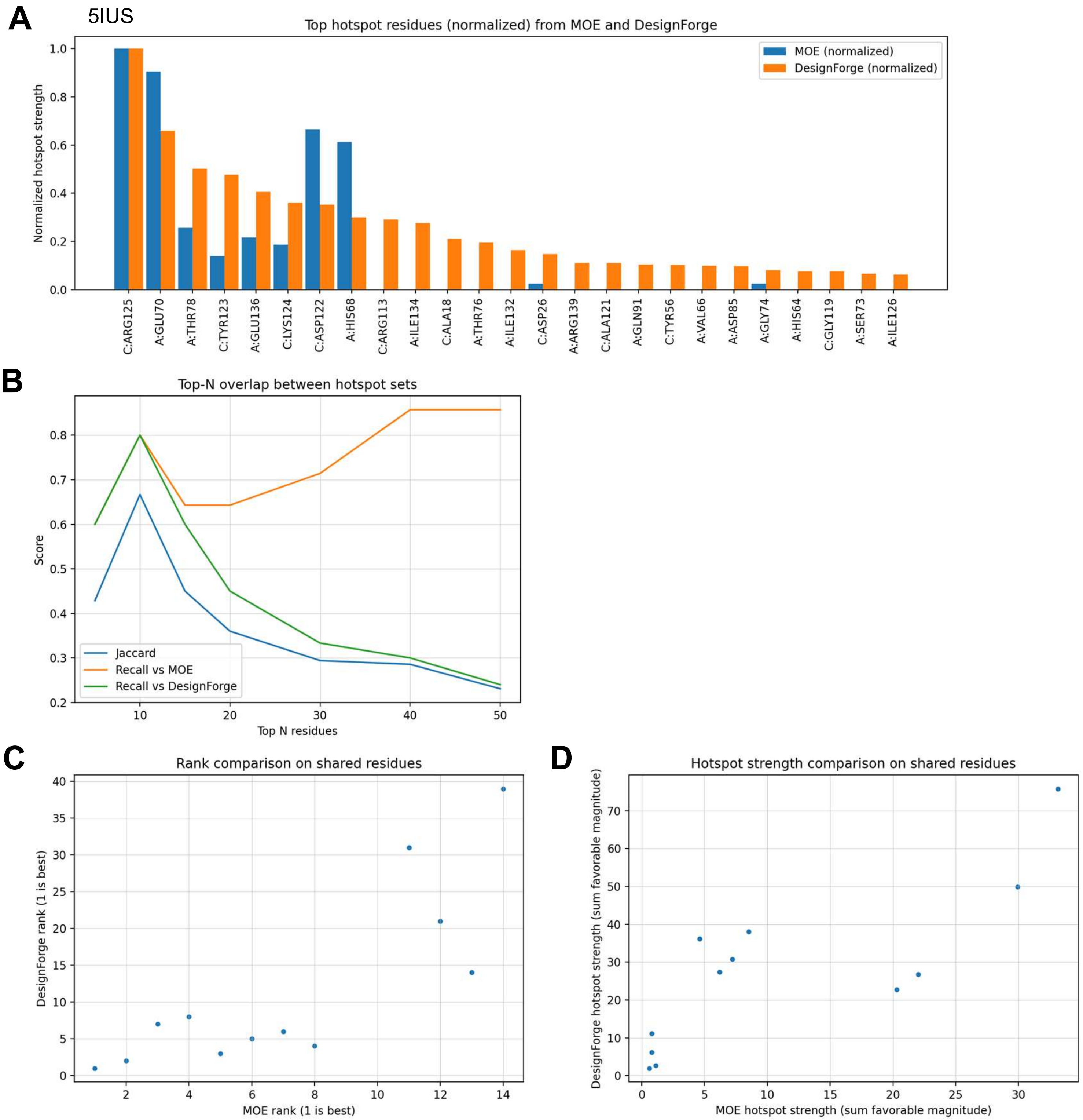
A
5IUS
Top hotspot residues (normalized) from MOE and DesignForge
MOE (normalized)
DesignForge (normalized)
Normalized hotspot strength
C:ARG125
A:GLU70
A:THR78
C:TYR123
A:GLU136
C:LYS124
C:ASP122
A:HIS68
C:ARG113
A:ILE134
C:ALA18
A:THR76
A:ILE132
C:ASP26
A:ARG139
C:ALA121
A:GLN91
C:TYR56
A:VAL66
A:ASP85
A:GLY74
A:HIS64
C:GLY119
A:SER73
A:ILE126
B
Top-N overlap between hotspot sets
Score
Top N residues
Jaccard
Recall vs MOE
Recall vs DesignForge
C
Rank comparison on shared residues
DesignForge rank (1 is best)
MOE rank (1 is best)
D
Hotspot strength comparison on shared residues
DesignForge hotspot strength (sum favorable magnitude)
MOE hotspot strength (sum favorable magnitude)

**Figure S2**

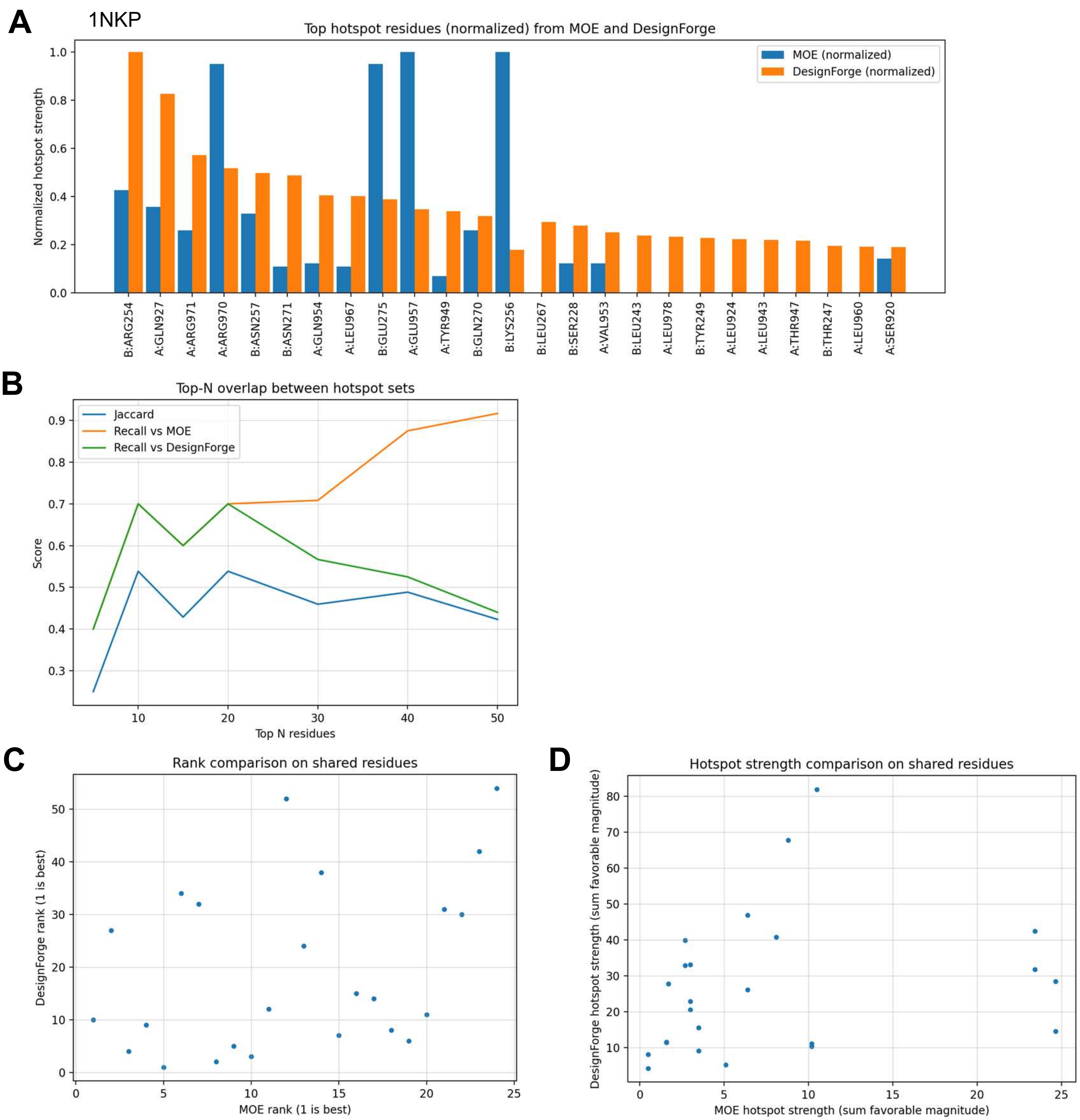
A
1NKP
Top hotspot residues (normalized) from MOE and DesignForge
MOE (normalized)
DesignForge (normalized)
Normalized hotspot strength
B:ARG254
A:GLN927
A:ARG971
A:ARG970
B:ASN257
B:ASN271
A:GLN954
A:LEU967
B:GLU275
A:GLU957
A:TYR949
B:GLN270
B:LYS256
B:LEU267
B:SER228
A:VAL953
B:LEU243
A:LEU978
B:TYR249
A:LEU924
A:LEU943
A:THR947
B:THR247
A:LEU960
A:SER920
B
Top-N overlap between hotspot sets
Jaccard
Recall vs MOE
Recall vs DesignForge
Score
Top N residues
C
Rank comparison on shared residues
DesignForge rank (1 is best)
MOE rank (1 is best)
D
Hotspot strength comparison on shared residues
DesignForge hotspot strength (sum favorable magnitude)
MOE hotspot strength (sum favorable magnitude)

**Figure S3**

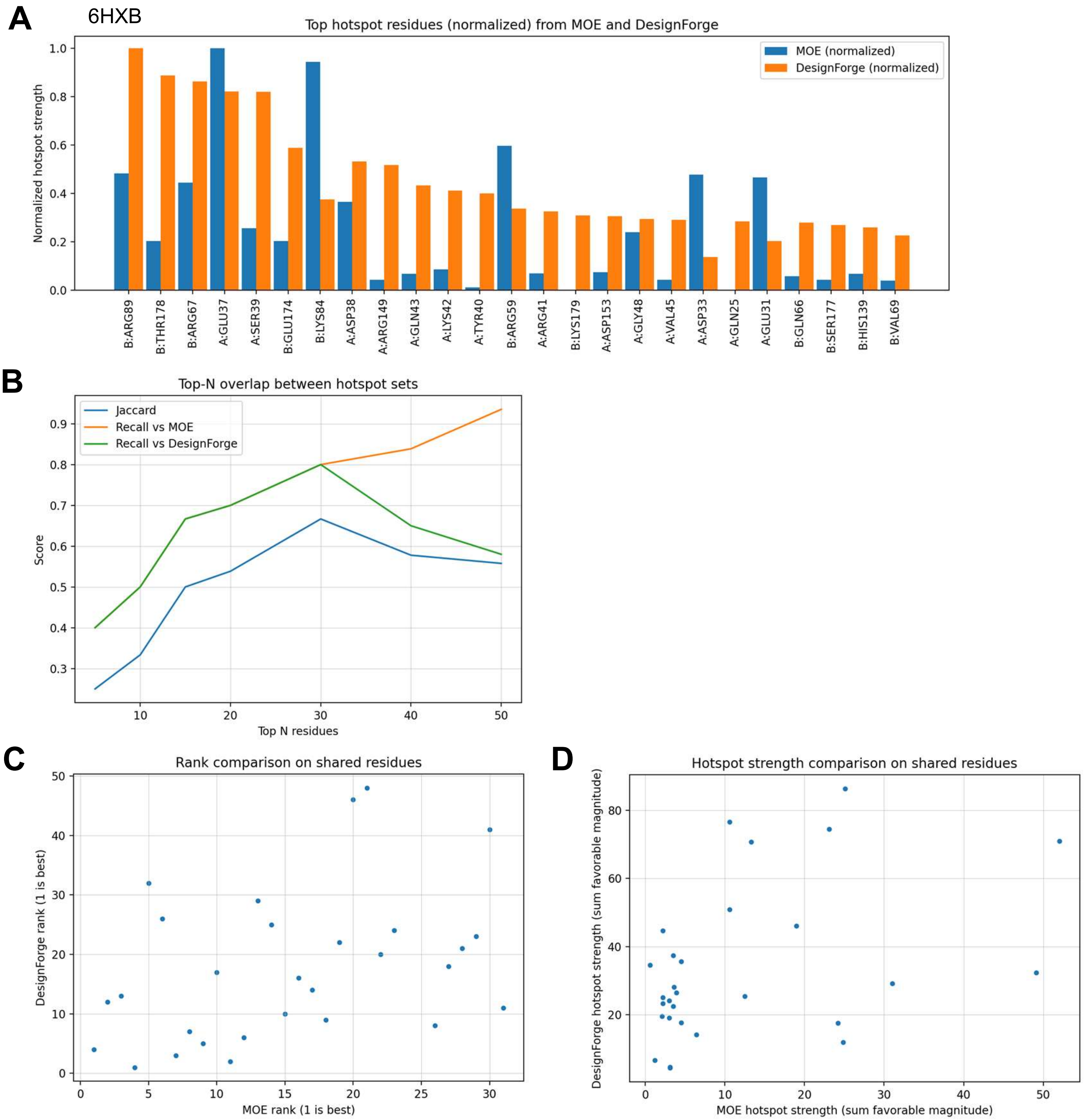

A
6HXB
Top hotspot residues (normalized) from MOE and DesignForge
MOE (normalized)
DesignForge (normalized)
Normalized hotspot strength
B:ARG89
B:THR178
B:ARG67
A:GLU37
A:SER39
B:GLU174
B:LYS84
A:ASP38
A:ARG149
A:GLN43
A:LYS42
A:TYR40
B:ARG59
A:ARG41
B:LYS179
A:ASP153
A:GLY48
A:VAL45
A:ASP33
A:GLN25
A:GLU31
B:GLN66
B:SER177
B:HIS139
B:VAL69
B
Top-N overlap between hotspot sets
Jaccard
Recall vs MOE
Recall vs DesignForge
Score
Top N residues
C
Rank comparison on shared residues
DesignForge rank (1 is best)
MOE rank (1 is best)
D
Hotspot strength comparison on shared residues
DesignForge hotspot strength (sum favorable magnitude)
MOE hotspot strength (sum favorable magnitude)


**Figure S4**

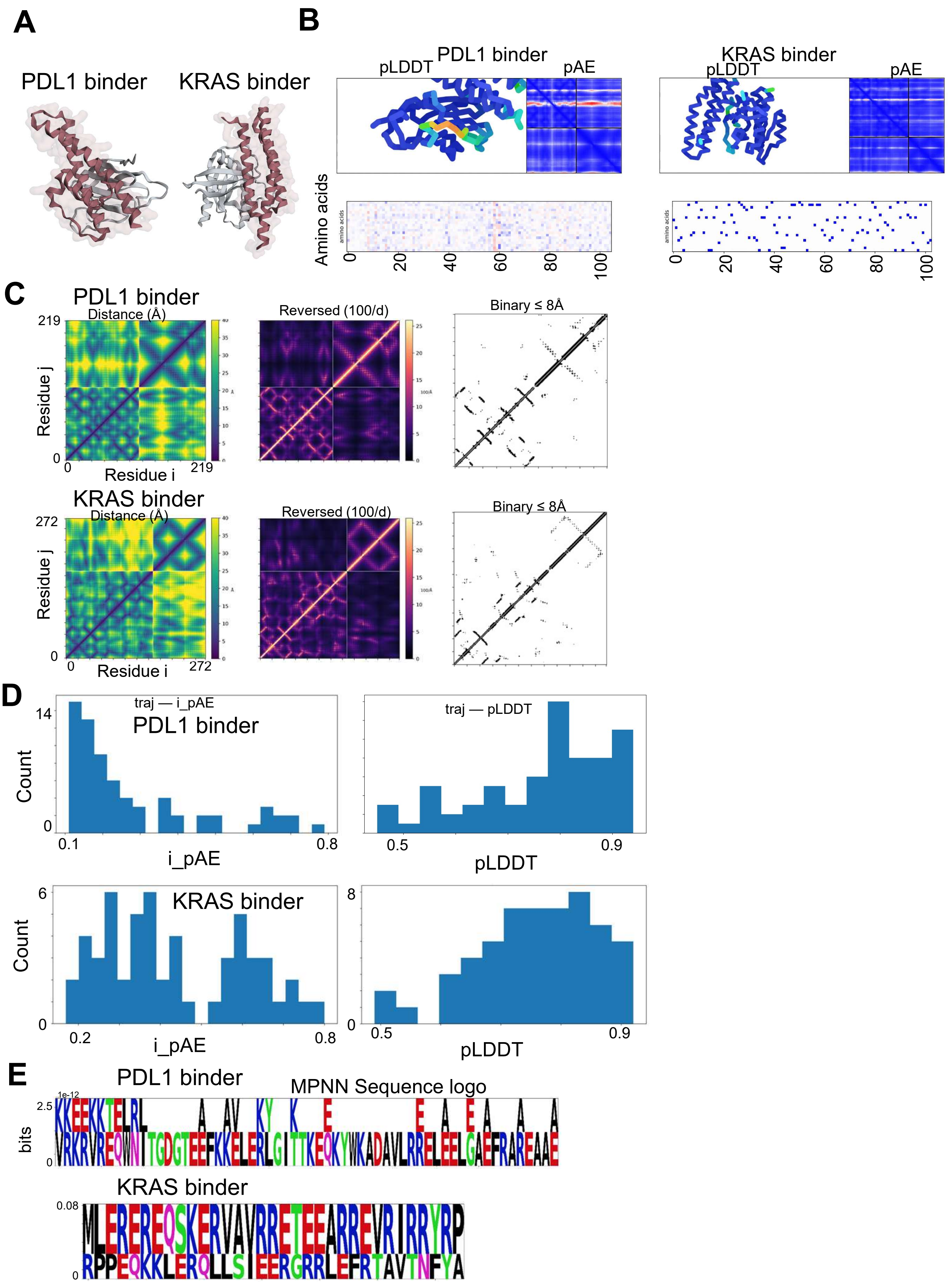
A
B
PDL1 binder
KRAS binder
pLDDT
pAE
Amino acids
C
PDL1 binder
Distance (Å)
Reversed (100/d)
Binary ≤ 8Å
Residue j
Residue i
KRAS binder
D
traj — i_pAE
traj — pLDDT
Count
i_pAE
pLDDT
E
MPNN Sequence logo
bits

Figure S5

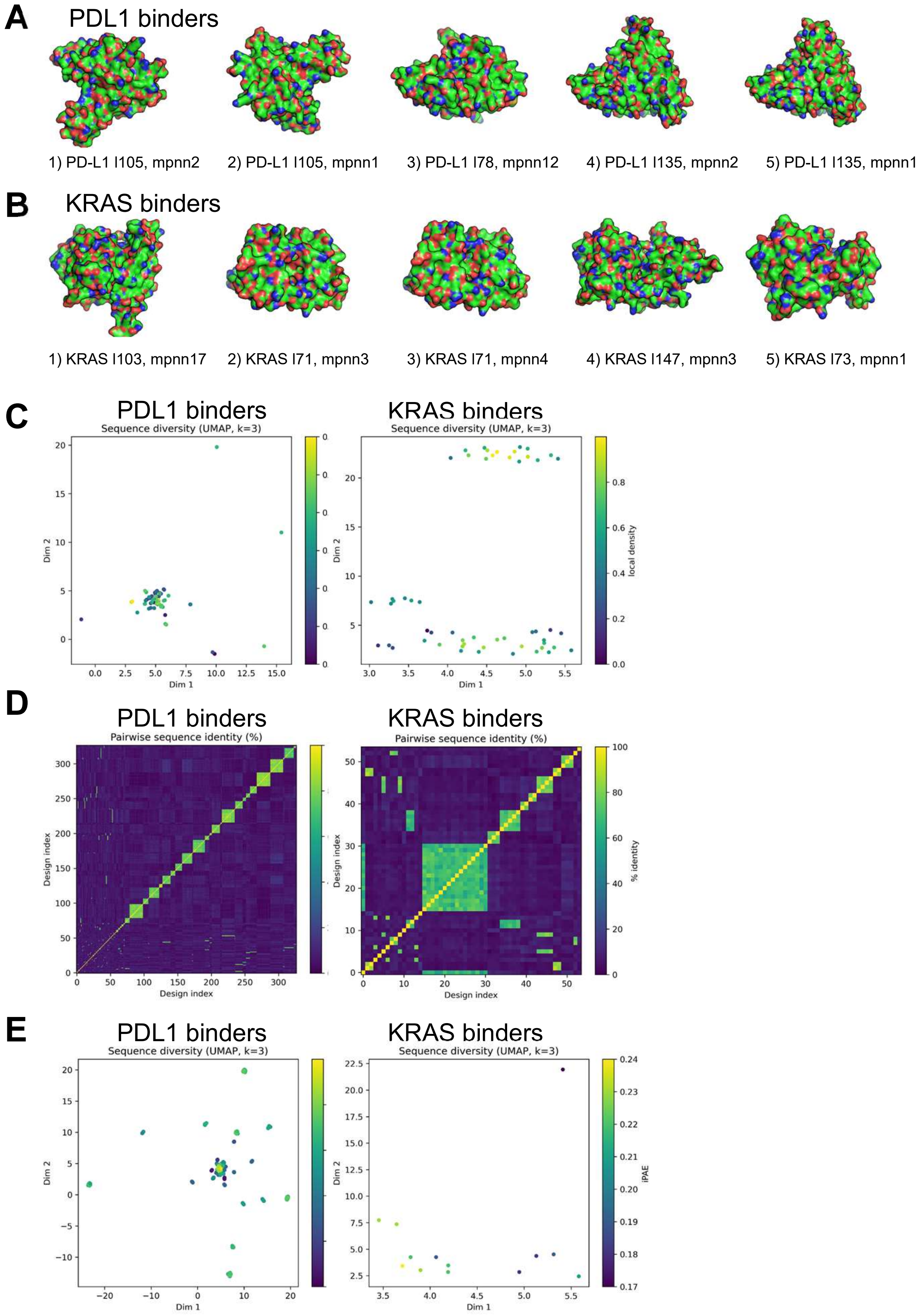
A
PDL1 binders
1) PD-L1 l105, mpnn2
2) PD-L1 l105, mpnn1
3) PD-L1 l78, mpnn12
4) PD-L1 l135, mpnn2
5) PD-L1 l135, mpnn1
B
KRAS binders
1) KRAS l103, mpnn17
2) KRAS l71, mpnn3
3) KRAS l71, mpnn4
4) KRAS l147, mpnn3
5) KRAS l73, mpnn1
C
PDL1 binders
Sequence diversity (UMAP, k=3)
Dim 1
Dim 2
KRAS binders
Sequence diversity (UMAP, k=3)
Dim 1
Dim 2
local density
D
PDL1 binders
Pairwise sequence identity (%)
Design index
Design index
KRAS binders
Pairwise sequence identity (%)
Design index
Design index
% identity
E
PDL1 binders
Sequence diversity (UMAP, k=3)
Dim 1
Dim 2
KRAS binders
Sequence diversity (UMAP, k=3)
Dim 1
Dim 2
iPAE

**Figure S6**

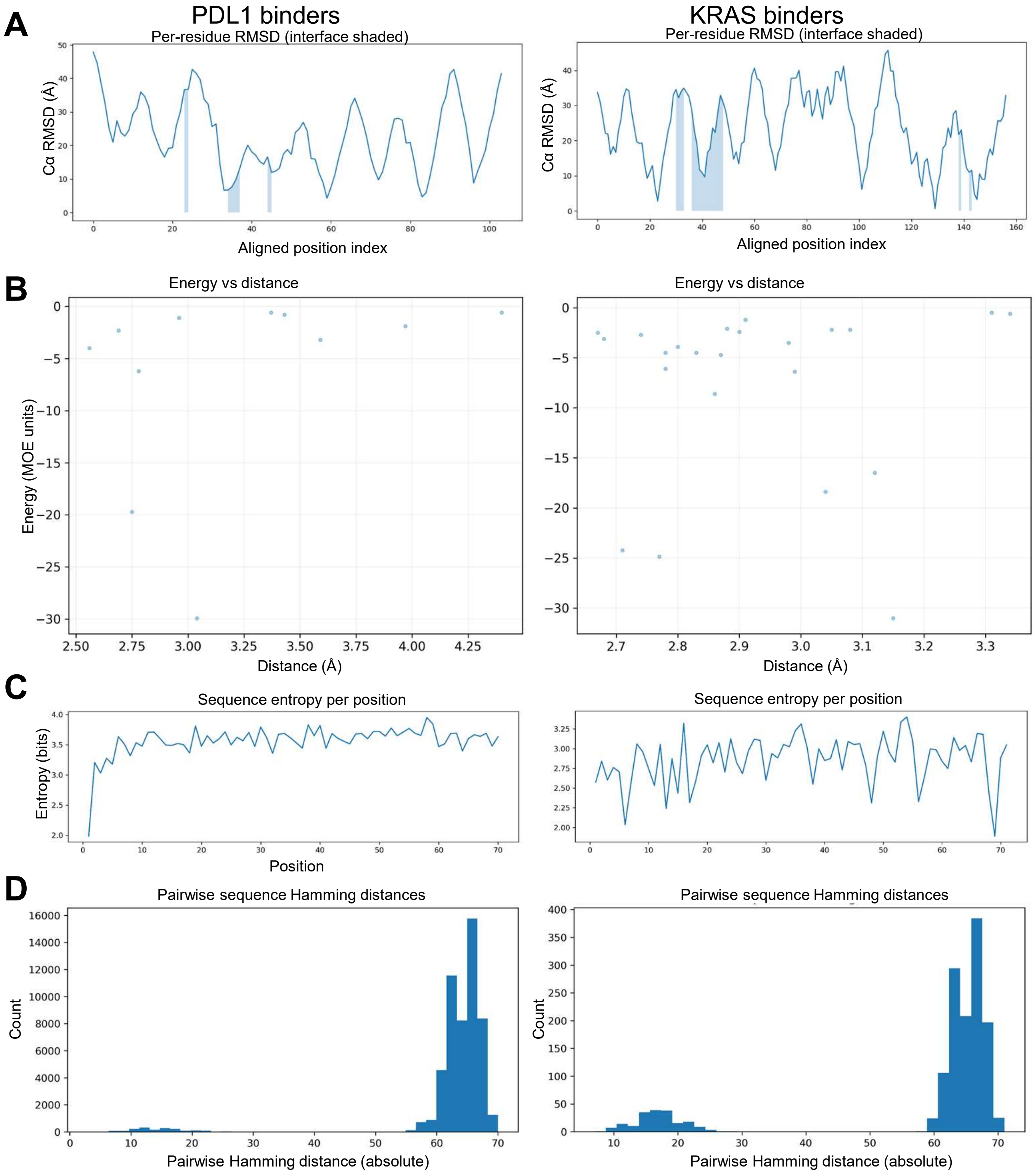

A
PDL1 binders
KRAS binders
Per-residue RMSD (interface shaded)
Cα RMSD (Å)
Aligned position index
B
Energy vs distance
Energy (MOE units)
Distance (Å)
C
Sequence entropy per position
Entropy (bits)
Position
D
Pairwise sequence Hamming distances
Count
Pairwise Hamming distance (absolute)


**Figure S7**
